# Self-rated eyesight among healthy older Australians: Baseline results of the ASPREE Longitudinal Study of Older Persons

**DOI:** 10.1111/ceo.14233

**Published:** 2023-04-28

**Authors:** Myra B. McGuinness, Liubov D. Robman, John J. McNeil, Cammie Tran, Robyn L. Woods, Alice J. Owen, Thao Pham, Robyn H. Guymer

**Affiliations:** 1 Centre for Eye Research Australia, Royal Victorian Eye & Ear Hospital, Melbourne, Australia; 2 Centre for Epidemiology and Biostatistics, Melbourne School of Population and Global Health, University of Melbourne, Melbourne, Australia; 3 Department of Epidemiology and Preventive Medicine, School of Public Health and Preventive Medicine, Monash University, Melbourne, Australia; 4 Department of Surgery (Ophthalmology), Melbourne Medical School, University of Melbourne, Melbourne, Australia

**Keywords:** epidemiology, population health, prevalence, self-assessment, vision impairment

## Abstract

**Background:**

We aimed to describe the self-reported level of eyesight amongst a cohort of relatively healthy older Australian adults, and to investigate associations between poorer self-rated eyesight and demographic, health, and functional characteristics

**Methods:**

The ASPirin in Reducing Events in the Elderly (ASPREE) Longitudinal Study of Older Persons (ALSOP) study was embedded in a multisite trial which recruited independently living Australians from general practices (2010–2014). Self-rated eyesight was recorded on a paper-based questionnaire as Excellent, Good, Fair, Poor, Very poor, or Completely blind at the baseline study wave

**Results:**

Data from 14 592 participants (aged 70–95 years, 54.61% female) were included in this cross-sectional analysis. Eighty percent of participants reported excellent or good eyesight (*n* = 11 677). People with complete blindness were precluded from enrolling but 299 participants (2.0%) reported poor or very poor eyesight, and 2616 rated their eyesight as fair (17.9%). Lower levels of eyesight were associated with being older, female, fewer years of formal education, a primary language other than English, smoking, and self-reported macular degeneration, glaucoma, retinopathy, cataracts, and hearing problems (each *p* ≤ 0.021). People with lower levels of eyesight had a higher number of falls, frailty characteristics, and depressive symptoms, and lower mental and physical health functioning scores (each *p* < 0.001)

**Conclusions:**

Whilst most of these healthy older Australians reported good or excellent eyesight, a notable minority reported poor or very poor eyesight, and this was associated with a range of poorer health measures. These findings support the need for additional resources to prevent vision loss and associated sequelae

## 1 ∣ INTRODUCTION

Recent estimates of the prevalence of vision impairment in Australia have been generated from population-based studies through formal measurement of visual acuity and visual fields.^1-5^ However, these are only two measures of the complex processes that contribute to visual function, and may not accurately represent the level of vision that an individual perceives themself to have. Given the recent focus on people-centred outcomes when planning for inclusivity and accessibility to community services and health care, an understanding of the level of self-reported visual function in the community is vital.^6^

Around 6.5% of older Australian adults are estimated to have vision impairment defined as presenting vision less than 6/12.^2^ The estimated prevalence of self-reported vision problems among older adults has ranged from almost 9% in North America to over 23% in Asia and Africa.^7-9^ Less information about self-reported visual impairment is available in Australia. The triennial National Health Survey administered by the Australian Bureau of Statistics estimated the prevalence of total and partial blindness to 2.3% of people over 70 years of age based on self-report.^10,11^ However, the definition of legal blindness in Australia (best corrected visual acuity worse than 6/60 or visual fields constricted within 10° of fixation in the better seeing eye) does not represent the full range of vision impairment present in the community.^12^ The burden of milder forms of visual impairment should also be considered when planning for resource allocation and service provision, especially given that even mild visual impairment can preclude driving and participation in other valued activities. In addition, quality of life is known to differ according to several aspects of vision that have not been used to gauge the prevalence of vision impairment in the past, such as contrast sensitivity, colour vision, glare, and binocular function.^13-15^ Therefore, a more general approach to capturing the level of visual function in the community is likely to be informative.

The ASPREE Longitudinal Study of Older Persons (ALSOP) aims to examine a broad range of factors relevant to the ageing Australian population.^16^ The baseline wave of this questionnaire-based cohort study captured participants' perspectives of their own level of vision. Using this data, we aimed to describe the distribution of - self-rated eyesight among generally healthy older Australians, and to investigate demographic, health, and functional characteristics associated with poorer self-rated eyesight.

## 2 ∣ METHODS

### 2.1 ∣ Study design

Demographic, lifestyle, and cognitive data were collected by study staff at baseline visits of the ASPirin in Reducing Events in the Elderly (ASPREE) randomised multicentre placebo-controlled trial (recruitment period 2010–2014, clinicaltrials.gov: NCT01038583), which evaluated the effect of 100 mg daily aspirin on physical disability-free and dementia-free survival.^17^ The paper-based, 14-section ALSOP Baseline Medical Questionnaire was mailed to Australian ASPREE participants within their first year of enrolment if they were still active in the study at that time.^16^

ASPREE was approved by Monash University Human Research Ethics Committee (HREC, 2006/745MC), RACGP Ethics Committee (NREEC 02/22b), University of Tasmania Ethics Committee (H0008933), Australian National University HREC (Protocol 2008/100), ACT Health HREC (ETH.11.07.997), and the University of Adelaide Ethics Committee (H-250-2011). ALSOP was approved by Monash University HREC (CF11/1100 and CF11/1935). Participants provided separate written informed consent for ASPREE and ALSOP. These studies were undertaken in accordance with the National Health and Medical Research Council of Australia Statement on Ethical Conduct in Human Research and conducted in accordance with the Declaration of Helsinki.

### 2.2 ∣ Recruitment and eligibility

Community-dwelling people aged ≥70 years were recruited into the ASPREE study through general practices across five south-eastern Australian states/territories. Exclusion criteria precluded enrolment of people with manifest cardiovascular disease, substantial disability, anaemia, high risk of bleeding, dementia, uncontrolled high blood pressure, or ongoing use of antiplatelet/anticoagulant medication.^17^ Individuals who needed assistance to complete basic activities of daily living (eating, dressing, walking across a room, bathing, toileting and transferring) were excluded. Participants were required to be able to read and sign a consent form as part of the eligibility criteria and the use of visual aids was permitted for these tasks. Participants were also informed that they would be required to complete selected written questionnaires confidentially without assistance from other individuals; potential participants who could not see well enough to read these forms (i.e., those with severe visual impairment) may have been less likely to participate.

### 2.3 ∣ Baseline ASPREE data

Demographic information (age at randomisation, gender, race, primary language, country of birth, years of formal education, living situation, area of residence) were collected from participants in person by study staff. Variables of interest were chosen a priori as potential predictors of eye disease.^7,18-21^ Postcode of residence was used to derive remoteness area (major city/not major city) and the Index of Relative Socio-economic Advantage and Disadvantage decile (1–5, lower advantage/higher disadvantage vs. 6–10, higher advantage/lower disadvantage).^22^ Health history included smoking and alcohol use (current/former/never). Disease status (present/absent) was derived for diabetes mellitus (self-report of diabetes, fasting glucose ≥7 mmol/L, or use of anti-hyperglycaemic medication), hypertension (blood pressure >140/90 mmHg, or on anti-hypertensive medication), and dyslipidaemia (serum cholesterol ≥5.5 mmol/L, low density lipoprotein >4.1 mmol/L, or use of cholesterol-lowering medication).^23^ Polypharmacy was defined as simultaneous use of ≥5 prescription medications. The 10-item Center for Epidemiological Studies depression scale (CES-D10, self-administered) was categorised as 0–7 (no/mild depressive symptoms), and 8–30 (depressive symptoms) in the previous week.^24,25^ Mental component and physical component quality-of-life scores were derived from the SF-12 (self-administered) and categorised in approximate quartile groupings.^26^ Frailty score was derived using modified Fried criteria from items relating to low body mass index, gait speed, grip strength, exhaustion, and low physical activity (assessed by study staff); individuals were categorised as frail (≥3 characteristics), pre-frail (1–2 characteristics), or non-frail.^27^

### 2.4 ∣ Eyesight section of ALSOP questionnaire

As worded in the validated National Eye Institute Visual Function Questionnaire,^28^ participants were asked, ‘At the present time, how would you rate your eyesight? (with glasses or contact lenses, if you wear them)’ Response options were Excellent, Good, Fair, Poor, Very poor, and Completely blind. Participants reported if a doctor had ever diagnosed them with ‘Macular degeneration’, ‘Glaucoma’, ‘Retinopathy or diabetic retinopathy’, or ‘Cataracts’ (response options Yes/No/Do not know). Those who reported a history of cataracts and cataract surgery were classified as no longer having cataracts. Difficulty performing the following vision-related activities was rated (no/little/some/extreme difficulty even when wearing glasses): ‘Recognising people when they are close’, ‘Watching television’, ‘Reading a newspaper or magazine’, ‘Reading small labels on food or medication’, and ‘Going up or down steps or a curb in dim light because of your eyesight’.

Participants were also asked ‘Do you have any problems with your hearing?’ (response options Yes/No/Do not know) in the hearing section of the ALSOP questionnaire.

### 2.5 ∣ Statistical methods

Participants with missing data on self-rated eyesight or demographics were excluded (i.e., complete-case analyses were conducted). The proportions of people in the target population with good/excellent, fair, and poor/very poor eyesight were estimated with exact binomial 95% confidence intervals (CIs).

The associations between self-rated eyesight (categorised as good/excellent, fair, very poor/poor, and completely blind) and demographic, health, and functional variables were investigated via multinomial logistic regression with adjustment for age and gender. The covariates were selected a priori as potential confounders of the exposure-eyesight associations and restricted to age and gender; these parsimonious models were chosen rather than making separate causal assumptions about confounding and mediation for every relationship of interest.^29^ Difficulty performing vision-related activities was compared according to self-rated eyesight status via Pearson's chi-squared test.

The initial sample size was chosen to power the ASPREE study's primary objectives. Because the primary objective of this paper is to describe the distribution of self-rated vision and not to test a single underlying hypothesis, no adjustment of significance levels has been performed for multiple-testing among these exploratory analyses.^30,31^ Analyses were conducted using Stata/MP v17.0 (College Station, TX).

## 3 ∣ RESULTS

### 3.1 ∣ Participant characteristics

**FIGURE 1:**
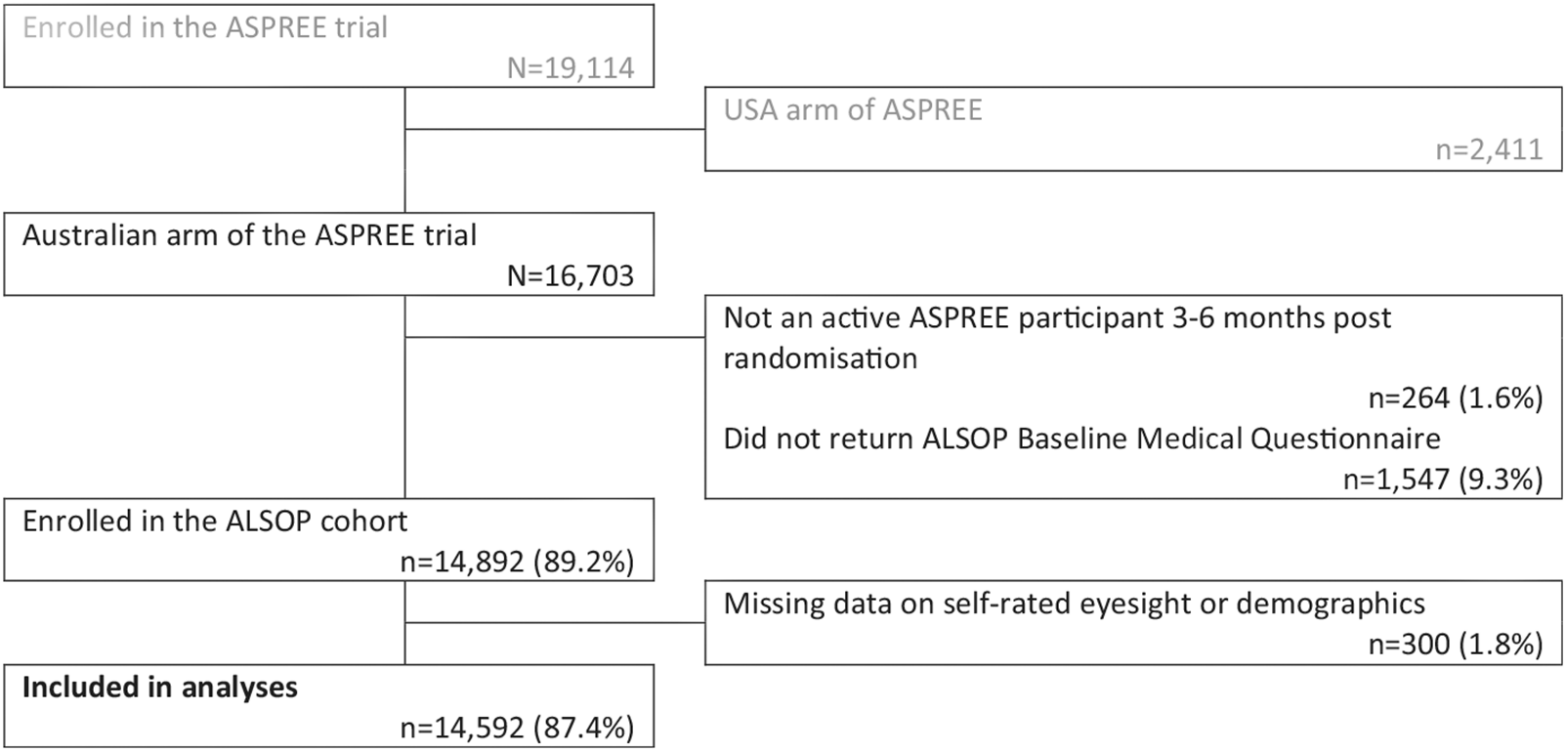
Study participation flow chart. ALSOP, ASPREE Longitudinal Study of Older Persons; ASPREE, ASPirin in Reducing Events in the Elderly trial.

Of the 16 703 Australian participants in the ASPREE trial, 2111 (12.6%) either did not participate in the ALSOP study or had missing eyesight or demographic data, leaving 14 592 participants in the analyses (87.4%, see Figure 1).

Included participants were aged 70–95 years (median 74.0, IQR 71.7–77.6) and 54.6% (*n* = 7969) were female (see Table 1). The majority were white (*n* = 14 411, 98.8%), spoke English as their primary language (*n* = 14 067, 96.4%), and were born in Australia (*n* = 11 016, 75.5%). Just over half lived in a major city (*n* = 7661, 52.5%) and in an area with higher advantage/lower disadvantage (*n* = 8313, 57.0%). Only 11 (0.1%) of the included participants were of Aboriginal or Torres Strait Islander descent. On average, the people enrolled in ALSOP who were excluded from the current study were slightly older, but other demographics were similarly distributed (see Table S1).

Most participants described their eyesight as good (*n* = 9399, 64.4%) or excellent (*n* = 2278, 15.6%; either good or excellent: 80.0%, 95% CI 79.4–80.7), and only 1.6% (*n* = 229) described their eyesight as poor and 0.5% (*n* = 70) described it as very poor (either poor or very poor: 2.0%, 95% CI 1.8–2.3). The remaining participants described their eyesight as fair (*n* = 2616, 17.9%, 95% CI 17.3–18.6). No participants described themselves as completely blind.

Participants with poor/very poor eyesight were more likely to be older, female, have a primary language other than English, and have fewer years of formal education (Table 1). Similar (but slightly lower) proportions of poor/very poor eyesight were observed among those living in a major city compared to those in regional areas, and among those residing in areas of higher advantage compared with those in lower-advantage areas.

### 3.2 ∣ Ocular history

Almost a third of all participants (*n* = 4091) reported they had undergone cataract surgery and 122 (3.0%) of these participants reported they still had poor/very poor vision. A similar proportion reporting poor/very poor vision was observed among those who reported having cataracts but no history of cataract surgery (80/2642, 3.0%).

**FIGURE 2:**
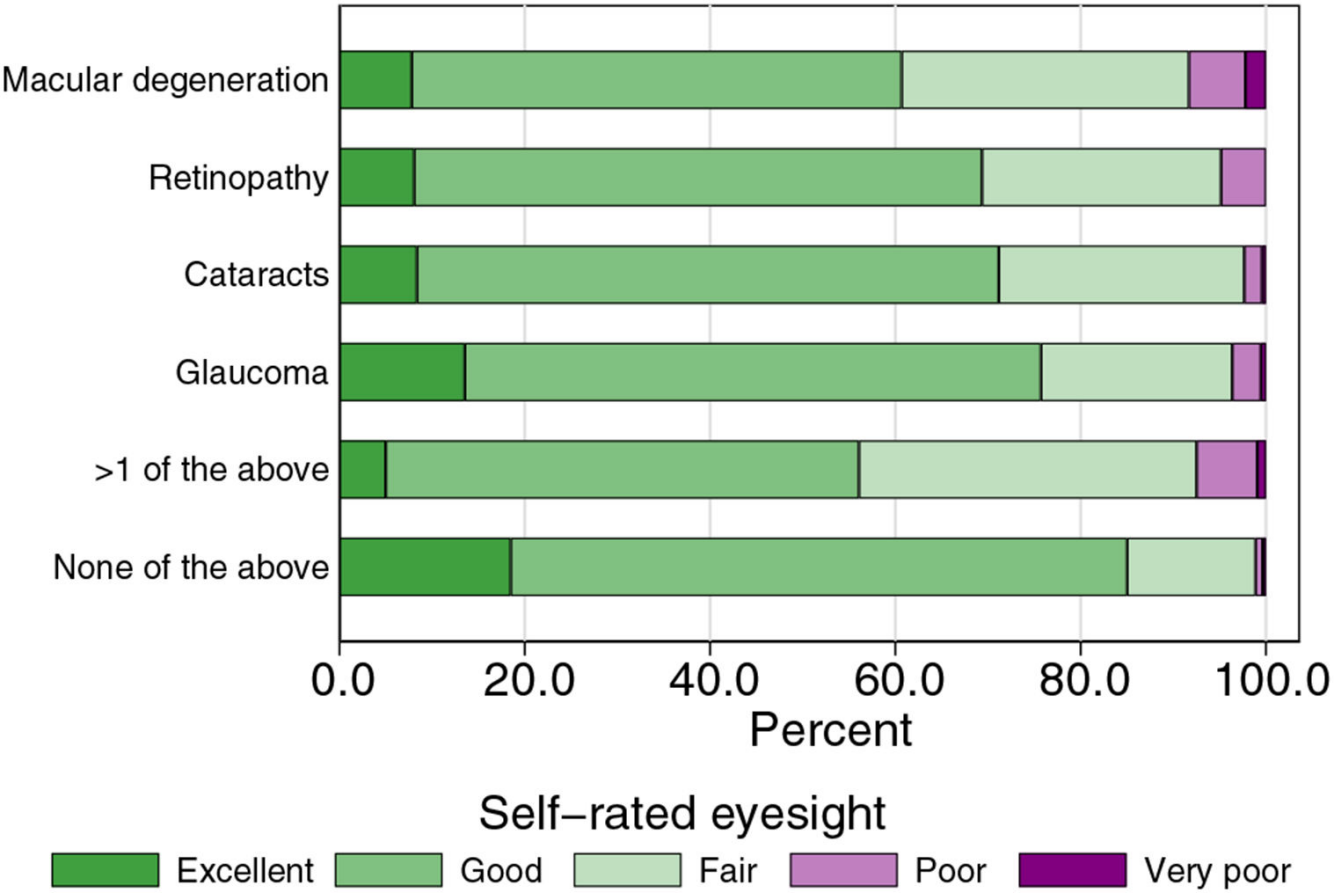
Distribution of self-rated eyesight according to self-reported ocular condition (*n* = 14 547; excludes 45 participants with missing data on history of ocular conditions).

Among the 928 participants who reported having a diagnosis of macular degeneration, 88 (9.5%) reported poor/very poor vision (including 39 people who also reported having another eye condition). Those reporting macular degeneration alone had greater odds of fair or poor/very poor eyesight compared to those with other eye conditions (see Table 2 and Figure 2).

Among the 1398 participants who were categorised as having diabetes mellitus, 65 (4.7%) reported retinopathy or diabetic retinopathy. Seventeen (26.2%) of these participants with diabetes and self-reported retinopathy reported fair eyesight, and six (9.2%) reported poor/very poor vision.

### 3.3 ∣ Self-rated eyesight and health history

Current smokers were more likely to report fair, poor or very poor eyesight compared to those who had never smoked, and former smokers were more likely to report fair eyesight than never-smokers (see Table 2).

Poor/very poor eyesight was associated with polypharmacy and self-rated hearing problems (<0.001 each, see Table 2). However, those with a history of dyslipidaemia were estimated to have 32% lower odds of poor/very poor eyesight compared to those without dyslipidaemia. Similar proportions of people were observed with a self-reported history of cataracts, macular degeneration, retinopathy/diabetic retinopathy, and glaucoma among those with dyslipidaemia or hearing problems compared to those without these conditions (see Table S2). People classified as having polypharmacy had higher proportions with a history of cataract (50.3%) or glaucoma (15.4%) compared to those with <5 simultaneous medications (39.6% and 7.2% respectively, see Table S2).

There was no evidence of an association between poor/very poor eyesight and any of diabetes mellitus, hypertension, or history of alcohol consumption (*p* ≥ 0.219, see Table 2).

### 3.4 ∣ Self-rated eyesight and visual, mental, and physical function

**FIGURE 3:**
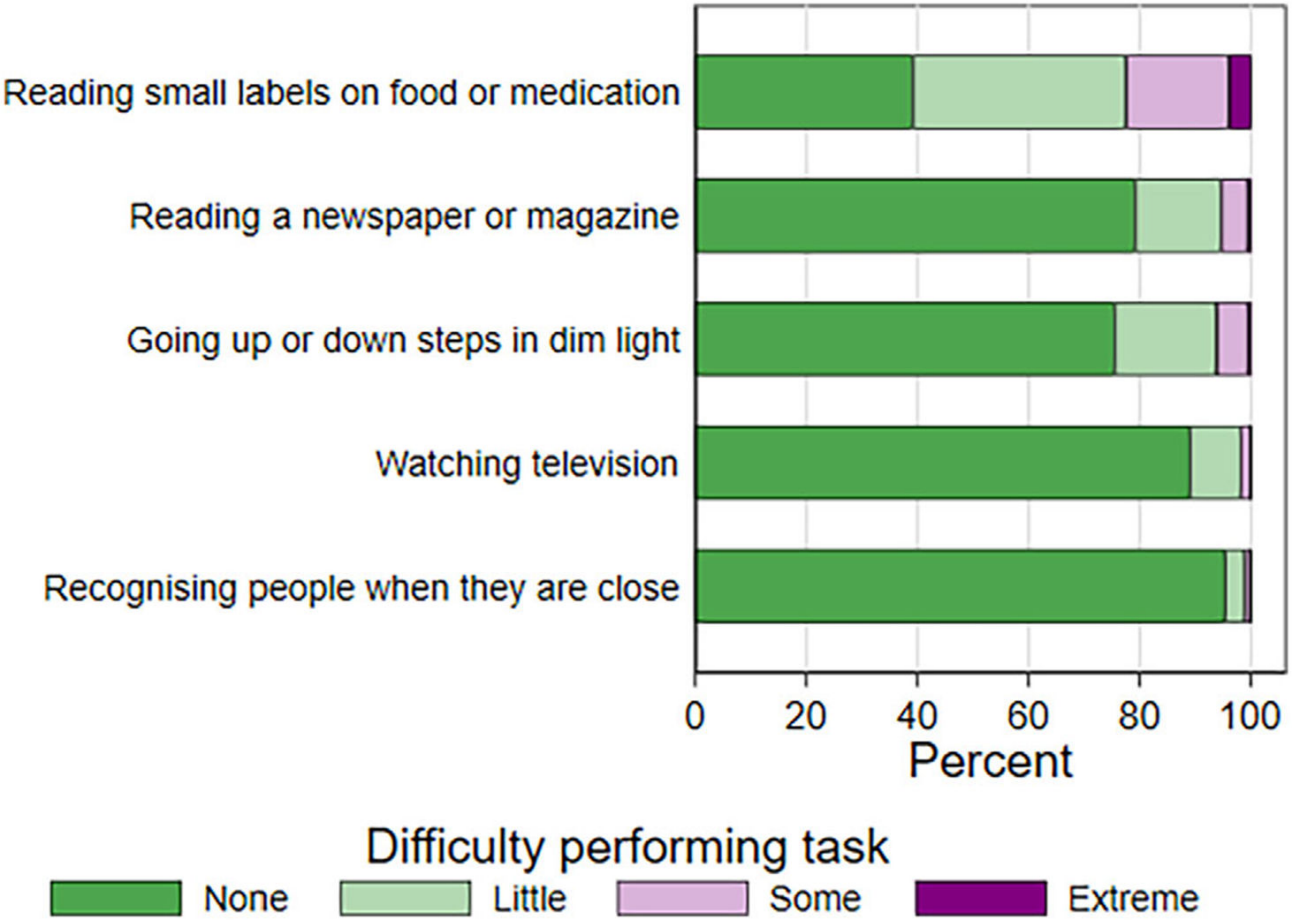
Distribution of difficulty performing vision-related activities.

Over half of all participants reported at least a little difficulty reading labels on food and medication (*n* = 8901, 61.0%, see Figure 3). However, most reported no difficulty recognising people when they are close (*n* = 13 840, 94.8%) or watching television (*n* = 12 893, 88.4%).

Self-rated eyesight was associated with difficulty performing each of the five vision-related activities that were presented (*p* < 0.001 each, see Table S3).

Participants with age-related macular degeneration or retinopathy tended to have more difficulty performing vision-related activities than participants who reported glaucoma or cataracts (see Figure S1 and Table S4).

Poorer levels of eyesight were observed, on average, as the number of frailty characteristics, depressive symptoms and falls increased, and with poorer SF-12 mental and physical component scores (see Table 3). Better mental and physical function was observed among participants who reported having none of the eye conditions of interest, and, on average, participants with self-reported retinopathy or diabetic retinopathy tended to have more depressive symptoms, more falls, and poorer SF-12 mental and physical component scores (see Table S5).

## 4 ∣ DISCUSSION

In this large cohort of generally healthy Australians aged 70 years and over, 80% reported having good or excellent eyesight. This high proportion bodes well for their ongoing engagement and participation in the community. However, >2% disclosed poor or very poor eyesight. The true prevalence of poor vision among this age group is likely to be higher in the wider community given that people with serious illness and severe disabilities (including blindness) were precluded from enrolment in this study.

We found higher proportions of people reporting poor eyesight among less advantaged groups such as those with fewer years of formal education and those with a primary language other than English. This suggests that access to eyecare may be more difficult for these groups, although uptake of healthcare services was not investigated in this study.^20,32,33^ Poorer levels of eyesight were associated with comorbidities such as depression, frailty, falls, and poorer SF-12 mental and physical scores, even after adjusting for age and gender. Thus, prevention or treatment of vision loss may play an important part in reducing the burden of a range of health issues.^6,34^ Smoking habits were also associated with poorer eyesight, reiterating the importance of a strong public health stance against cigarette use.

Encouragingly, two-thirds of the participants who reported having glaucoma, age-related macular degeneration or retinopathy stated that their eyesight was either good or excellent. This may, in part, reflect early detection and treatment of these eye conditions among this cohort. Although less than half the Australian population aged 70 years or above had private health insurance when the baseline study wave of ALSOP was conducted, two-thirds of the participants who enrolled in the study were insured.^16,35^ This suggests a proactive approach to healthcare which may have contributed to the good levels of vision reported among those with ocular conditions. The poorest visual outcomes were observed among those with age-related macular degeneration. These findings underline the need for further resourcing to be directed towards research, detection, and treatment for macular disease. Treatment for neovascular macular degeneration had only been available to Australians in the decade prior to the baseline study wave of ALSOP, and hence would not have benefited those with long standing disease or those with the atrophic form of macular degeneration.

Although a self-reported history of retinopathy or diabetic retinopathy was associated with poorer eyesight, a diagnosis of diabetes was not. The prevalence of self-reported retinopathy among diabetics in this study was lower than expected in Australia.^36^ This may be explained in part by the exclusion of individuals with manifest cardiovascular disease, a known risk factor for diabetic retinopathy.^37^ This discrepancy is likely to have diluted the estimated association between diabetes and poor eyesight. In addition, a major contributor to underreported diabetic retinopathy in Australia is the high number of undiagnosed cases.^38^ Hence, figures based on self-report are likely to underestimate its true prevalence.^38^

Most people did not report trouble recognising faces or watching TV, although those reporting poorer eyesight were more likely to have difficulty with these tasks. Conversely, over half of all participants reported difficulty reading labels on food and medication, suggesting that improving the legibility of these labels would benefit a large proportion of the older population. In addition, issues with seeing in dim light, especially when negotiating steps, is an important consideration when planning community spaces.

We found people reporting reduced eyesight were also more likely to report hearing problems, even after adjusting for age. Dual sensory impairment (i.e., concurrent hearing and vision impairment) is not uncommon among people in this age group.^21^ A higher proportion of people with hearing problems reported macular degeneration and glaucoma compared to those who did not report hearing problems.

### 4.1 ∣ Comparison to previous finding

The 2017–2018 Australian National Health Survey estimated the prevalence of self-reported partial or total blindness to be 2.3% amongst people over 70 years of age,^10,11^ similar to the proportion in this study who reported their eyesight to be poor or very poor. Given ASPREE participants were required to complete selected written forms without assistance from other individuals, it is unsurprising that none of the people in the current study reported themselves as ‘Completely blind’. It is difficult to compare to prevalence of self-reported vision problems in other countries given the differences in questionnaire wording that those findings have been based upon. However, these estimates have ranged from 9% in the United States to over 23% in Nigeria and in China.^7-9^

In the mid-1990s, it was estimated that 8.3% of Victorians aged 65 years and above had bilateral visual impairment as determined by presenting visual acuity (<6/12) and/or reduced visual field.^1^ One third of these cases were attributable to uncorrected refractive error.^1^ In the late 1990s, 2.1% of participants of the Blue Mountains Eye Study II (aged 49–98 years) were found to have visual acuity <6/12 which did not improve with optical correction, and this was associated with poorer total, mental, and physical SF-36 scores.^3^ This supports the association between self-rated eyesight and SF-12 scores observed in the current study. More recently, >10% of non-indigenous participants aged ≥70 years in the National Eye Health Survey were found to have bilateral vision impairment (presenting vision <6/12), with higher levels among the Indigenous community.^2^ A high proportion of these cases was attributed to uncorrected refractive error.^39^ In the current study, participants were asked to rate their eyesight while wearing optical correction, which may explain some of the discrepancy between the prevalence of visual impairment reported in previous studies and that of poor self-rated eyesight in this study, and suggests that many people with correctable refractive error alone do not consider their eyesight to be ‘Poor’. Only a small number of people in the current study identified as Aboriginal or Torres Strait Islander. Thus, we cannot comment on differences between Indigenous and non-indigenous Australians.

Like previous studies, we found that poor vision was associated with an increased number of falls.^6^ Several aspects of visual function such as depth perception, contrast sensitivity and peripheral vision combine to assist in the avoidance of tripping hazards and improvement of balance.^40-43^ Given the risk of mortality and the economic burden resulting from falls, the prevention and treatment of eye disease is likely to have an important impact across several health indices related to falls.^34^ Our research also highlights the need to create safe and accessible environments for older Australians with diminished vision, including the provision of good lighting.

Debate continues on the role of elevated lipid levels and statin use in the development and progression of age-related macular degeneration and cataracts.^44,45^ In this study, we found participants with dyslipidaemia (defined by medication use and/or blood biochemistry) had lower odds of poor eyesight but there was no substantial difference in the prevalence of eye conditions according to dyslipidaemia status. It was beyond the scope of this paper to investigate the associations between eye diseases and the types of lipid-lowering medication being taken.

### 4.2 ∣ Strengths and limitations

Strengths of this study include the large sample size in metropolitan and regional areas. ALSOP had a high questionnaire completion rate, and the age distribution of participants was broadly alike that of the wider community.^16,17^ However, our study is limited by not including people with serious health, functional, or cognitive issues. Bias towards the selection of people with better visual function for inclusion in the study is likely due to the barriers to enrolment in the ASPREE faced by people with severe visual disability. People born in Australia and speaking English as a primary language were overrepresented in this study compared to the same age group in the wider population.^11^ Thus, the results of this study cannot be generalised to the entire population of older adult Australians. In addition, we expect that the prevalence of poor eyesight to be greater in remote communities and those with large Indigenous populations.^2,46^

Self-rated eyesight was assessed via the ‘General vision’ item from the widely used National Eye Institute Visual Function Questionnaire.^28^ It has previously been shown to be correlated with visual acuity, near vision, visual fields, and contrast sensitivity.^13,14,47^ However, its responses cannot be directly mapped to any one measure of visual function given an individual's perception of their own eyesight is likely to be influenced by their occupational and recreational needs, their past experiences, and the multiple processes that are simultaneously required for good visual function (e.g., ocular motility, stereopsis/fusion, dark/light adaptation, accommodation, colour discrimination, etc.). In addition, no visual function tests were performed during the current study, so we are unable to verify the reliability of the scale in this population.

The breadth of data from ALSOP and ASPREE allowed multiple aspects of health to be examined. However, due to the cross-sectional nature of this analysis, no comment on the direction of causality can be made. It is plausible that poor vision could lead to future events such as falls and depression.^6^ However, there are shared predictors of poor general health and some ocular conditions,^19,48^ and it is also possible that people with depressive symptoms perceive their vision to be worse than when experiencing better levels of mental health.^49^ In addition, estimates comparing eyesight between subgroups may be biased due the lack of control for confounding variables other than age and gender. In particular, smoking is a known risk factor for eye disease and several of the health and mental conditions investigated.^19^ However, our primary aim was to describe the distribution of eyesight within the subgroups, rather than to quantify the proportion of vision impairment directly attributable to each co-morbidity.

Categorisations of ocular disease were based on self-report and are subject to misclassification bias.^38^

### 4.3 ∣ Future research

Follow-up data from the second and third waves of ALSOP will provide more evidence about the direction of causality between poor eyesight and other characteristics of interest.^16^ In future analyses, data from the ASPREE age-related macular degeneration sub-study may be used to investigate in detail the relationship between dyslipidaemia, use of lipids and the prevalence and incidence of retinal conditions.^50^ Additional research is needed to assess the prevalence of poor eyesight amongst minority groups including Indigenous Australians and adults with serious illnesses.

### 4.4 ∣ Conclusions

The majority of healthy older participants in this study reported good or excellent eyesight. However, less advantaged groups were at higher risk for poorer eyesight, reinforcing the need for healthcare services that are equally accessible for all members of society. Associations were detected between poorer levels of eyesight and other health issues. These findings support the need for additional preventative health measures for those with poor vision. Consideration of the needs of people with poorer vision should be given to facilitate their full engagement in the community.

## Supplementary Material

Supplementary Material

## Figures and Tables

**TABLE 1:** Association between self-rated eyesight and participant characteristics.

		Self-rated vision							
		Fair (*n* = 2616)				Poor/very poor (*n* = 299)			
	*N*	*n* (row %)	RRR	[95% CI]	*p*	*n* (row %)	RRR	[95% CI]	*p*
Age (years)									
70–74	8541	1385 (16.2%)	1.00			130 (1.5%)	1.00		
75–79	3843	745 (19.4%)	1.26	[1.14,1.39]	<0.001	90 (2.3%)	1.60	[1.22,2.10]	0.001
80–84	1677	356 (21.2%)	1.42	[1.25,1.62]	<0.001	51 (3.0%)	2.15	[1.55,2.99]	<0.001
85–95	531	130 (24.5%)	1.77	[1.44,2.18]	<0.001	28 (5.3%)	4.03	[2.64,6.14]	<0.001
Gender									
Male	6623	1203 (18.2%)	1.00			114 (1.7%)	1.00		
Female	7969	1413 (17.7%)	0.97	[0.89,1.06]	0.472	185 (2.3%)	1.32	[1.04,1.67]	0.021
Race									
White	14 411	2574 (17.9%)	1.00			293 (2.0%)	1.00		
Aboriginal	11	4 (36.4%)	2.69	[0.78,9.24]	0.115	0 (0.0%)	NA		
Asian	103	22 (21.4%)	1.33	[0.83,2.14]	0.241	4 (3.9%)	2.35	[0.85,6.48]	0.100
>1 race/other	67	16 (23.9%)	1.51	[0.86,2.66]	0.155	2 (3.0%)	1.84	[0.44,7.62]	0.403
Primary language									
English	14 067	2500 (17.8%)	1.00			279 (2.0%)	1.00		
Not English	525	116 (22.1%)	1.35	[1.09,1.67]	0.006	20 (3.8%)	2.18	[1.36,3.47]	0.001
Country of birth									
Australia	11 016	1970 (17.9%)	1.00			219 (2.0%)	1.00		
Overseas	3576	646 (18.1%)	1.02	[0.92,1.13]	0.688	80 (2.2%)	1.17	[0.91,1.52]	0.226
Years of education									
<9	2436	532 (21.8%)	1.00			77 (3.2%)	1.00		
9–12	6392	1120 (17.5%)	0.77	[0.68,0.86]	<0.001	126 (2.0%)	0.62	[0.46,0.82]	0.001
>12	5764	964 (16.7%)	0.73	[0.65,0.82]	<0.001	96 (1.7%)	0.54	[0.40,0.74]	<0.001
Living situation									
At home alone	4570	901 (19.7%)	1.00			103 (2.3%)	1.00		
With family/friends	9975	1706 (17.1%)	0.88	[0.80,0.96]	0.006	194 (1.9%)	1.07	[0.83,1.39]	0.581
Retirement home	47	9 (19.1%)	0.91	[0.44,1.90]	0.802	2 (4.3%)	1.54	[0.36,6.58]	0.559
IRSAD decile									
1–5 (Less advantage)	6279	1198 (19.1%)	1.00			137 (2.2%)	1.00		
6–10 (More advantage)	8313	1418 (17.1%)	0.87	[0.80,0.95]	0.002	162 (1.9%)	0.88	[0.69,1.10]	0.258
State/territory									
ACT	672	107 (15.9%)	1.00			14 (2.1%)	1.00		
New South Wales	1054	195 (18.5%)	1.18	[0.91,1.53]	0.206	23 (2.2%)	1.07	[0.54,2.09]	0.853
South Australia	1294	189 (14.6%)	0.88	[0.68,1.14]	0.325	19 (1.5%)	0.65	[0.33,1.32]	0.236
Tasmania	1817	336 (18.5%)	1.19	[0.94,1.51]	0.158	42 (2.3%)	1.12	[0.61,2.07]	0.714
Victoria	9755	1789 (18.3%)	1.17	[0.94,1.44]	0.158	201 (2.1%)	0.98	[0.57,1.70]	0.952
Lives in a major city									
No	6931	1293 (18.7%)	1.00			148 (2.1%)	1.00		
Yes	7661	1323 (17.3%)	0.91	[0.83,0.99]	0.022	151 (2.0%)	0.90	[0.71,1.13]	0.354

Abbreviations: ACT, Australian Capital Territory; IRSAD, Index of Relative Socio-economic Advantage and Disadvantage; RRR, relative-risk ratio estimated via multinomial logistic regression with good/excellent self-rated vision as the base outcome category, adjusted for age as a continuous variable and gender (coefficients for age category adjusted for gender only).

**TABLE 2:** Association between self-rated eyesight and health history (*n* = 14 592).

		Self-rated vision							
		Fair (*n* = 2616)				Poor/very poor (*n* = 299)			
	*N*	*n* (row %)	RRR	[95% CI]	*p*	*n* (row %)	RRR	[95% CI]	*p*
Eye condition^a^									
None of these	10 200	1414 (13.9%)	1.00			114 (1.1%)	1.00		
Current cataracts	2143	568 (26.5%)	2.33	[2.08,2.61]	<0.001	50 (2.3%)	2.47	[1.76,3.46]	<0.001
Glaucoma	910	188 (20.7%)	1.61	[1.35,1.91]	<0.001	33 (3.6%)	3.25	[2.19,4.84]	<0.001
Macular degeneration	590	183 (31.0%)	2.96	[2.45,3.58]	<0.001	49 (8.3%)	8.67	[6.07,12.40]	<0.001
Retinopathy	62	16 (25.8%)	2.17	[1.22,3.87]	0.009	3 (4.8%)	5.28	[1.60,17.37]	0.006
>1 of the above	642	234 (36.4%)	3.94	[3.31,4.69]	<0.001	48 (7.5%)	9.41	[6.59,13.43]	<0.001
Missing	45	13 (28.9%)				2 (4.4%)			
Cataract surgery									
No	10 394	1908 (18.4%)	1.00			170 (1.6%)	1.00		
Yes	4091	683 (16.7%)	0.79	[0.71,0.87]	<0.001	122 (3.0%)	1.35	[1.04,1.74]	0.022
Do not know	14	5 (35.7%)	2.33	[0.76,7.20]	0.140	1 (7.1%)	4.76	[0.58,39.09]	0.146
Missing	93	20 (21.5%)				6 (6.5%)			
Smoking status									
Never	8132	1378 (16.9%)	1.00			157 (1.9%)	1.00		
Former	6010	1133 (18.9%)	1.16	[1.06,1.27]	0.001	123 (2.0%)	1.23	[0.96,1.57]	0.107
Current	450	105 (23.3%)	1.62	[1.29,2.03]	<0.001	19 (4.2%)	2.98	[1.82,4.89]	<0.001
Alcohol status									
Never	2320	415 (17.9%)	1.00			56 (2.4%)	1.00		
Former	688	150 (21.8%)	1.31	[1.06,1.62]	0.013	18 (2.6%)	1.32	[0.76,2.28]	0.319
Current	11 584	2051 (17.7%)	1.01	[0.89,1.13]	0.915	225 (1.9%)	0.92	[0.68,1.24]	0.584
Hypertension									
No	3703	647 (17.5%)	1.00			74 (2.0%)	1.00		
Yes	10 889	1969 (18.1%)	1.01	[0.91,1.11]	0.854	225 (2.1%)	0.96	[0.73,1.25]	0.749
Diabetes mellitus									
No	13 194	2382 (18.1%)	1.00			276 (2.1%)	1.00		
Yes	1398	234 (16.7%)	0.89	[0.77,1.03]	0.128	23 (1.6%)	0.76	[0.50,1.17]	0.219
Dyslipidaemia									
No	4729	882 (18.7%)	1.00			119 (2.5%)	1.00		
Yes	9863	1734 (17.6%)	0.94	[0.86,1.03]	0.205	180 (1.8%)	0.68	[0.54,0.87]	0.002
Polypharmacy									
No	10 910	1860 (17.0%)	1.00			185 (1.7%)	1.00		
Yes	3682	756 (20.5%)	1.26	[1.15,1.39]	<0.001	114 (3.1%)	1.77	[1.39,2.25]	<0.001
Hearing problems									
No	7582	1106 (14.6%)	1.00			126 (1.7%)	1.00		
Yes	6525	1407 (21.6%)	1.60	[1.46,1.74]	<0.001	161 (2.5%)	1.60	[1.25,2.03]	<0.001
Do not know	387	90 (23.3%)	1.81	[1.42,2.32]	<0.001	9 (2.3%)	1.63	[0.82,3.24]	0.166
Missing	98	13 (13.3%)				3 (3.1%)			

Abbreviations: *N*, total number in category, *n* (%), number and row percentage with fair or poor/very poor vision; RRR, relative-risk ratio estimated via multinomial logistic regression with good/excellent self-rated vision as the base outcome category, adjusted for age as a continuous variable and gender.

a Eye conditions are self-reported.

**TABLE 3:** Association between self-rated eyesight and mental and physical function (*n* = 14 592).

		Self-rated vision							
		Fair (*n* = 2616)				Poor/very poor (*n* = 299)			
	*N*	*n* (row %)	RRR	[95% CI]	*p*	*n* (row %)	RRR	[95% CI]	*p*
CES-D10 depression score									
0–7 (no/mild symptoms)	13 230	2262 (17.1%)	1.00			238 (1.8%)	1.00		
8–30 (more symptoms)	1358	352 (25.9%)	1.76	[1.55,2.01]	<0.001	61 (4.5%)	2.81	[2.10,3.76]	<0.001
Missing	4	2 (50.0%)				0 (0.0%)			
Mental component score^a^									
≥60 (better function)	4342	710 (16.4%)	1.00			75 (1.7%)	1.00		
57–<60	3782	534 (14.1%)	0.85	[0.76,0.97]	0.012	56 (1.5%)	0.88	[0.62,1.25]	0.481
52–<57	3016	597 (19.8%)	1.28	[1.14,1.45]	<0.001	64 (2.1%)	1.30	[0.93,1.83]	0.126
<52 (poorer function)	3447	773 (22.4%)	1.52	[1.35,1.70]	<0.001	104 (3.0%)	1.89	[1.40,2.56]	<0.001
Missing	5	2 (40.0%)				0 (0.0%)			
Physical component score^a^									
≥55 (better function)	3918	528 (13.5%)	1.00			55 (1.4%)	1.00		
50–<55	3624	563 (15.5%)	1.16	[1.02,1.32]	0.025	44 (1.2%)	0.84	[0.56,1.25]	0.391
43–<50	3477	677 (19.5%)	1.54	[1.35,1.74]	<0.001	91 (2.6%)	1.80	[1.28,2.54]	0.001
<43 (poorer function)	3568	846 (23.7%)	1.96	[1.74,2.22]	<0.001	109 (3.1%)	2.10	[1.50,2.93]	<0.001
Missing	5	2 (40.0%)				0 (0.0%)			
Frailty score									
Not frail	9142	1512 (16.5%)	1.00			138 (1.5%)	1.00		
Pre-frail	5213	1036 (19.9%)	1.20	[1.09,1.31]	<0.001	148 (2.8%)	1.68	[1.32,2.14]	<0.001
Frail	237	68 (28.7%)	1.88	[1.40,2.53]	<0.001	13 (5.5%)	3.12	[1.70,5.72]	<0.001
Number of falls									
None in past year	10 253	1692 (16.5%)	1.00			172 (1.7%)	1.00		
1 in past year	2224	402 (18.1%)	1.10	[0.97,1.24]	0.123	47 (2.1%)	1.16	[0.84,1.61]	0.375
2 in past year	1187	268 (22.6%)	1.49	[1.28,1.72]	<0.001	34 (2.9%)	1.77	[1.22,2.58]	0.003
3 in past year	499	134 (26.9%)	1.85	[1.51,2.28]	<0.001	19 (3.8%)	2.34	[1.43,3.81]	0.001
4 or more in past year	287	85 (29.6%)	2.30	[1.76,2.99]	<0.001	20 (7.0%)	5.24	[3.21,8.54]	<0.001
Missing	142	35 (24.6%)				7 (4.9%)			

Abbreviations: *N*, total number in category, *n* (%), number and row percentage with fair or poor/very poor vision; RRR, relative-risk ratio estimated via multinomial logistic regression with good/excellent self-rated vision as the base outcome category, adjusted for age as a continuous variable and gender.

a Derived from SF-12 questionnaire.
