# Supplementary material for: Self-rated eyesight among healthy older Australians: Baseline results of the ASPREE Longitudinal Study of Older Persons
Source: Clin Exp Ophthalmol. Author manuscript; Available in PMC 2024 Jul 1. (PMC10348348; doi:10.1111/ceo.14233)
Supplement: Supplementary Material [file NIHMS1912920-supplement-Supplementary_Material.docx]

**Supporting Information**

Self-rated eyesight among healthy older Australians:

baseline results of the ASPREE Longitudinal Study of Older Persons

Supplementary Table 1: Demographic characteristics of ASPREE Longitudinal Study of Older Persons participants included and excluded from analyses

|  | **Included in analyses** | | **Total** | **p**† |
| --- | --- | --- | --- | --- |
|  | **No** | **Yes** |  |  |
|  | n=300 | n=14,592 | N=14,892 |  |
| Age at randomisation (years), n (%) |  |  |  |  |
| 70-74 | 135 (45.0%) | 8,541 (58.5%) | 8,676 (58.3%) |  |
| 75-79 | 84 (28.0%) | 3,843 (26.3%) | 3,927 (26.4%) |  |
| 80-84 | 60 (20.0%) | 1,677 (11.5%) | 1,737 (11.7%) |  |
| 85-95 | 21 ( 7.0%) | 531 ( 3.6%) | 552 ( 3.7%) |  |
| Gender, n (%) |  |  |  | 0.479 |
| Male | 130 (43.3%) | 6,623 (45.4%) | 6,753 (45.3%) |  |
| Female | 170 (56.7%) | 7,969 (54.6%) | 8,139 (54.7%) |  |
| Race, n (%) |  |  |  | 0.546 |
| White | 288 (96.0%) | 14,411 (98.8%) | 14,699 (98.7%) |  |
| Aboriginal/Torres Strait Islander | 0 ( 0.0%) | 11 ( 0.1%) | 11 ( 0.1%) |  |
| Asian | 3 ( 1.0%) | 103 ( 0.7%) | 106 ( 0.7%) |  |
| More than one race/Other | 2 ( 0.7%) | 67 ( 0.5%) | 69 ( 0.5%) |  |
| Missing | 7 ( 2.3%) | 0 ( 0.0%) | 7 ( 0.0%) |  |
| Primary language, n (%) |  |  |  | 0.807 |
| English | 290 (96.7%) | 14,067 (96.4%) | 14,357 (96.4%) |  |
| Not English | 10 ( 3.3%) | 525 ( 3.6%) | 535 ( 3.6%) |  |
| Country of birth |  |  |  | 0.096 |
| Australia | 239 (79.7%) | 11,016 (75.5%) | 11,255 (75.6%) |  |
| Overseas | 61 (20.3%) | 3,576 (24.5%) | 3,637 (24.4%) |  |
| Years of education, n (%) |  |  |  | 0.077 |
| < 9 | 64 (21.3%) | 2,436 (16.7%) | 2,500 (16.8%) |  |
| 9-12 | 129 (43.0%) | 6,392 (43.8%) | 6,521 (43.8%) |  |
| >12 | 106 (35.3%) | 5,764 (39.5%) | 5,870 (39.4%) |  |
| Missing | 1 ( 0.3%) | 0 ( 0.0%) | 1 ( 0.0%) |  |
| Living situation, n (%) |  |  |  | 0.118 |
| At home alone | 97 (32.3%) | 4,570 (31.3%) | 4,667 (31.3%) |  |
| At home with family/friends/spouse | 200 (66.7%) | 9,975 (68.4%) | 10,175 (68.3%) |  |
| In a residential/retirement home | 3 ( 1.0%) | 47 ( 0.3%) | 50 ( 0.3%) |  |
| IRSAD decile, n (%) |  |  |  | 0.582 |
| 1-5 (lower levels of advantage) | 115 (38.3%) | 6,279 (43.0%) | 6,394 (42.9%) |  |
| 6-10 (higher levels of advantage) | 142 (47.3%) | 8,313 (57.0%) | 8,455 (56.8%) |  |
| Missing | 43 (14.3%) | 0 ( 0.0%) | 43 ( 0.3%) |  |
| State/territory of residence, n (%) |  |  |  | 0.554 |
| Australian Capital Territory | 7 ( 2.3%) | 672 ( 4.6%) | 679 ( 4.6%) |  |
| New South Wales | 17 ( 5.7%) | 1,054 ( 7.2%) | 1,071 ( 7.2%) |  |
| South Australia | 26 ( 8.7%) | 1,294 ( 8.9%) | 1,320 ( 8.9%) |  |
| Tasmania | 36 (12.0%) | 1,817 (12.5%) | 1,853 (12.4%) |  |
| Victoria | 171 (57.0%) | 9,755 (66.9%) | 9,926 (66.7%) |  |
| Missing | 43 (14.3%) | 0 ( 0.0%) | 43 ( 0.3%) |  |
| Lives in a major city, n (%) |  |  |  | 0.381 |
| No | 115 (38.3%) | 6,931 (47.5%) | 7,046 (47.3%) |  |
| Yes | 142 (47.3%) | 7,661 (52.5%) | 7,803 (52.4%) |  |
| Missing | 43 (14.3%) | 0 ( 0.0%) | 43 ( 0.3%) |  |
| Self-reported eyesight, n (%) |  |  |  | 0.579 |
| Excellent/good | 40 (13.3%) | 11,677 (80.0%) | 11,717 (78.7.3%) |  |
| Fair | 8 ( 2.7%) | 2,616 (17.9%) | 2,624 (17.6%) |  |
| Poor/very poor | 0 ( 0.0%) | 299 ( 2.0%) | 299 ( 2.0%) |  |
| Missing | 252 (84.0%) | 0 ( 0.0%) | 252 ( 1.7%) |  |
| IRSAD = index of relative socio-economic advantage and disadvantage † p-values from Fisher's exact test (race, living situation, self-reported eyesight) and Pearson's chi-squared test (all other variables) | | | | |

Supplementary Table 2: Self-reported eye conditions according to dyslipidaemia, hearing and polypharmacy status

|  | **Total** | **Dyslipidaemia‡** | | **Self-reported  hearing problems** | | **Polypharmacy‡** | |
| --- | --- | --- | --- | --- | --- | --- | --- |
|  |  | **No** | **Yes** | **No/don't know†** | **Yes** | **No** | **Yes** |
|  | (N=14,547†) | (n=4,711) | (n=9,836) | (n=7,948) | (n=6,502) | (n=10,877) | (n=3,670) |
| Cataract history |  |  |  |  |  |  |  |
| Never | 7,653 (52.6%) | 2,542 (54.0%) | 5,111 (52.0%) | 4,290 (54.0%) | 3,324 (51.1%) | 6,069 (55.8%) | 1,584 (43.2%) |
| Ever | 6,147 (42.3%) | 1,955 (41.5%) | 4,192 (42.6%) | 3,240 (40.8%) | 2,861 (44.0%) | 4,302 (39.6%) | 1,845 (50.3%) |
| Don't know | 264 ( 1.8%) | 82 ( 1.7%) | 182 ( 1.9%) | 152 ( 1.9%) | 109 ( 1.7%) | 193 ( 1.8%) | 71 ( 1.9%) |
| Missing | 483 ( 3.3%) | 132 ( 2.8%) | 351 ( 3.6%) | 266 ( 3.3%) | 208 ( 3.2%) | 313 ( 2.9%) | 170 ( 4.6%) |
| Lens status |  |  |  |  |  |  |  |
| Never had cataracts diagnosed | 7,306 (50.2%) | 2,441 (51.8%) | 4,865 (49.5%) | 4,103 (51.6%) | 3,166 (48.7%) | 5,843 (53.7%) | 1,463 (39.9%) |
| Cataract not yet extracted | 2,642 (18.2%) | 812 (17.2%) | 1,830 (18.6%) | 1,410 (17.7%) | 1,218 (18.7%) | 1,872 (17.2%) | 770 (21.0%) |
| Had cataract extraction | 4,091 (28.1%) | 1,313 (27.9%) | 2,778 (28.2%) | 2,146 (27.0%) | 1,904 (29.3%) | 2,791 (25.7%) | 1,300 (35.4%) |
| Don't know | 256 ( 1.8%) | 77 ( 1.6%) | 179 ( 1.8%) | 146 ( 1.8%) | 107 ( 1.6%) | 187 ( 1.7%) | 69 ( 1.9%) |
| Missing | 252 ( 1.7%) | 68 ( 1.4%) | 184 ( 1.9%) | 143 ( 1.8%) | 107 ( 1.6%) | 184 ( 1.7%) | 68 ( 1.9%) |
| Macular degeneration |  |  |  |  |  |  |  |
| No | 12,133 (83.4%) | 3,960 (84.1%) | 8,173 (83.1%) | 6,710 (84.4%) | 5,351 (82.3%) | 9,263 (85.2%) | 2,870 (78.2%) |
| Yes | 928 ( 6.4%) | 290 ( 6.2%) | 638 ( 6.5%) | 465 ( 5.9%) | 455 ( 7.0%) | 635 ( 5.8%) | 293 ( 8.0%) |
| Don't know | 311 ( 2.1%) | 122 ( 2.6%) | 189 ( 1.9%) | 138 ( 1.7%) | 170 ( 2.6%) | 214 ( 2.0%) | 97 ( 2.6%) |
| Missing | 1,175 ( 8.1%) | 339 ( 7.2%) | 836 ( 8.5%) | 635 ( 8.0%) | 526 ( 8.1%) | 765 ( 7.0%) | 410 (11.2%) |
| Retinopathy/diabetic retinopathy | |  |  |  |  |  |  |
| No | 12,200 (83.9%) | 3,968 (84.2%) | 8,232 (83.7%) | 6,743 (84.8%) | 5,384 (82.8%) | 9,298 (85.5%) | 2,902 (79.1%) |
| Yes | 127 ( 0.9%) | 47 ( 1.0%) | 80 ( 0.8%) | 65 ( 0.8%) | 62 ( 1.0%) | 66 ( 0.6%) | 61 ( 1.7%) |
| Don't know | 539 ( 3.7%) | 195 ( 4.1%) | 344 ( 3.5%) | 244 ( 3.1%) | 291 ( 4.5%) | 384 ( 3.5%) | 155 ( 4.2%) |
| Missing | 1,681 (11.6%) | 501 (10.6%) | 1,180 (12.0%) | 896 (11.3%) | 765 (11.8%) | 1,129 (10.4%) | 552 (15.0%) |
| Glaucoma |  |  |  |  |  |  |  |
| No | 11,854 (81.5%) | 3,873 (82.2%) | 7,981 (81.1%) | 6,542 (82.3%) | 5,240 (80.6%) | 9,156 (84.2%) | 2,698 (73.5%) |
| Yes | 1,352 ( 9.3%) | 436 ( 9.3%) | 916 ( 9.3%) | 701 ( 8.8%) | 640 ( 9.8%) | 788 ( 7.2%) | 564 (15.4%) |
| Don't know | 259 ( 1.8%) | 100 ( 2.1%) | 159 ( 1.6%) | 123 ( 1.5%) | 134 ( 2.1%) | 187 ( 1.7%) | 72 ( 2.0%) |
| Missing | 1,082 ( 7.4%) | 302 ( 6.4%) | 780 ( 7.9%) | 582 ( 7.3%) | 488 ( 7.5%) | 746 ( 6.9%) | 336 ( 9.2%) |
| † Excludes 45 people with missing data on all eye conditions. 98 people with missing response on hearing included with those who responded No or Don't know. ‡ Polypharmacy defined as simultaneous use of ≥5 medications. Dyslipidaemia defined as serum cholesterol ≥5.5mmol/L, low density lipoprotein >4.1 mmol/L, or use of cholesterol-lowering medication. | | | | | | | |

Supplementary Table 3: Association between self-rated eyesight and difficulty performing vision-related activities

| **Difficulty** | **Self-rated vision**, n (%) | | |  |
| --- | --- | --- | --- | --- |
|  | **Excellent/Good** | **Fair** | **Poor/**  **Very poor** | **Total** |
|  | (n=11,677) | (n=2,616) | (n=299) | (N=14,592) |
| Recognising people when they are close |  |  |  |  |
| None | 11,335 (97.1%) | 2,319 (88.6%) | 186 (62.2%) | 13,840 (94.8%) |
| Little | 247 ( 2.1%) | 202 ( 7.7%) | 63 (21.1%) | 512 ( 3.5%) |
| Some | 40 ( 0.3%) | 70 ( 2.7%) | 36 (12.0%) | 146 ( 1.0%) |
| Extreme | 2 ( 0.0%) | 1 ( 0.0%) | 8 ( 2.7%) | 11 ( 0.1%) |
| Missing | 53 ( 0.5%) | 24 ( 0.9%) | 6 ( 2.0%) | 83 ( 0.6%) |
| Watching television |  |  |  |  |
| None | 10,908 (93.4%) | 1,864 (71.3%) | 121 (40.5%) | 12,893 (88.4%) |
| Little | 633 ( 5.4%) | 581 (22.2%) | 105 (35.1%) | 1,319 ( 9.0%) |
| Some | 63 ( 0.5%) | 136 ( 5.2%) | 62 (20.7%) | 261 ( 1.8%) |
| Extreme | 2 ( 0.0%) | 2 ( 0.1%) | 2 ( 0.7%) | 6 ( 0.0%) |
| Missing | 71 ( 0.6%) | 33 ( 1.3%) | 9 ( 3.0%) | 113 ( 0.8%) |
| Reading a newspaper or magazine |  |  |  |  |
| None | 10,061 (86.2%) | 1,301 (49.7%) | 70 (23.4%) | 11,432 (78.3%) |
| Little | 1,284 (11.0%) | 886 (33.9%) | 68 (22.7%) | 2,238 (15.3%) |
| Some | 223 ( 1.9%) | 363 (13.9%) | 110 (36.8%) | 696 ( 4.8%) |
| Extreme | 12 ( 0.1%) | 26 ( 1.0%) | 47 (15.7%) | 85 ( 0.6%) |
| Missing | 97 ( 0.8%) | 40 ( 1.5%) | 4 ( 1.3%) | 141 ( 1.0%) |
| Reading small labels on food or medication |  |  |  |  |
| None | 5,237 (44.8%) | 422 (16.1%) | 24 ( 8.0%) | 5,683 (38.9%) |
| Little | 4,557 (39.0%) | 969 (37.0%) | 59 (19.7%) | 5,585 (38.3%) |
| Some | 1,686 (14.4%) | 921 (35.2%) | 94 (31.4%) | 2,701 (18.5%) |
| Extreme | 165 ( 1.4%) | 286 (10.9%) | 118 (39.5%) | 569 ( 3.9%) |
| Missing | 32 ( 0.3%) | 18 ( 0.7%) | 4 ( 1.3%) | 54 ( 0.4%) |
| Going up or down steps in dim light |  |  |  |  |
| None | 9,395 (80.5%) | 1,476 (56.4%) | 89 (29.8%) | 10,960 (75.1%) |
| Little | 1,838 (15.7%) | 757 (28.9%) | 75 (25.1%) | 2,670 (18.3%) |
| Some | 386 ( 3.3%) | 337 (12.9%) | 98 (32.8%) | 821 ( 5.6%) |
| Extreme | 12 ( 0.1%) | 31 ( 1.2%) | 33 (11.0%) | 76 ( 0.5%) |
| Missing | 46 ( 0.4%) | 15 ( 0.6%) | 4 ( 1.3%) | 65 ( 0.4%) |
| p-value <0.001 for each activity (Pearson's chi-squared test) | | | | |

| 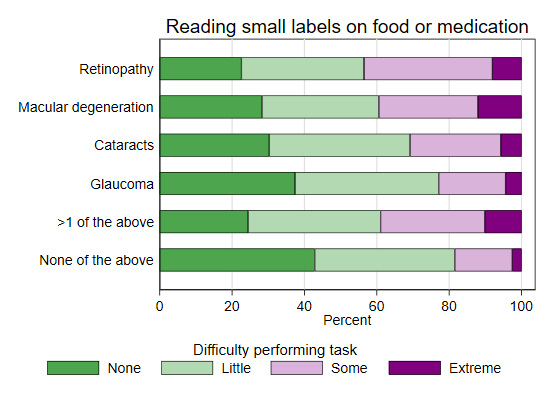 | 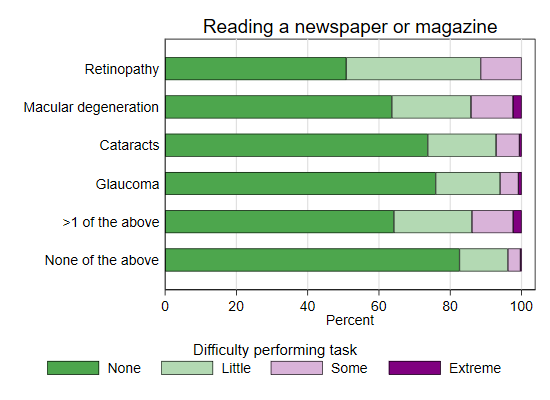 |
| --- | --- |
| 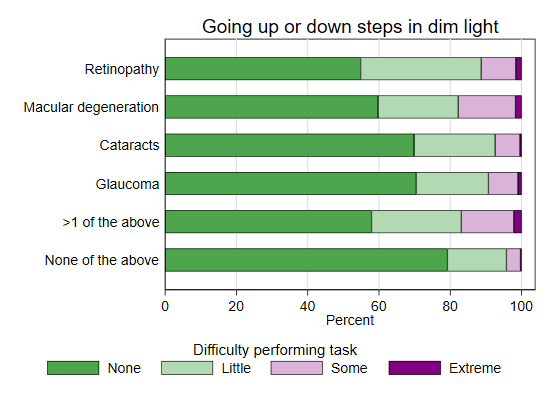 | 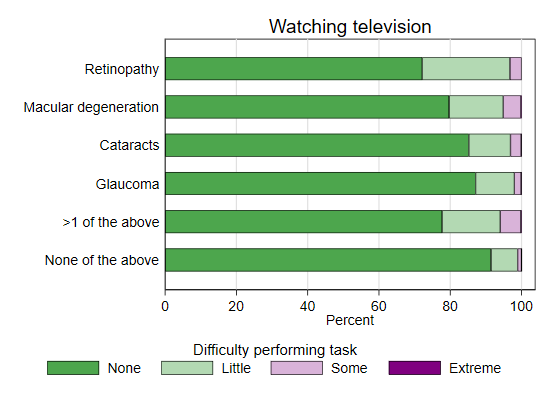 |
| 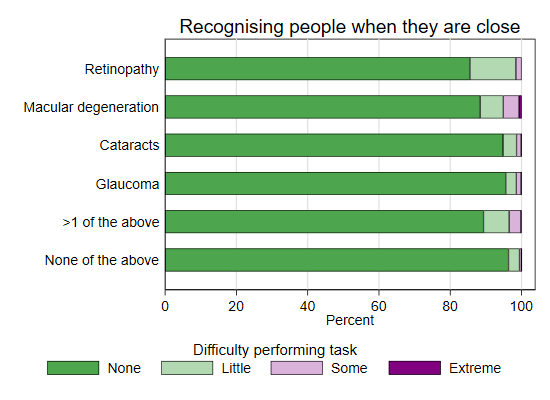 |  |

Supplementary Figure 1: Difficulty performing visual activities according to self-reported eye conditions. Percentage from number with non-missing activity data for each condition.

Supplementary Table 4: Difficulty performing visual activities according to self-reported eye condition (n=14,547, excludes 45 participants with missing data on eye conditions)

|  | **Self-reported ocular condition**, n (%) | | | | | |
| --- | --- | --- | --- | --- | --- | --- |
|  | **None of these** | **Cataracts** | **Glaucoma** | **Macular  degeneration** | **Retinopathy** | **>1 of these** |
|  | n=10200 | n=2143 | n=2143 | n=590 | n=62 | n=642 |
| Difficulty recognising people |  |  |  |  |  |  |
| No difficulty | 9788 (96.0%) | 2019 (94.2%) | 860 (94.5%) | 519 (88.0%) | 53 (85.5%) | 569 (88.6%) |
| Little difficulty | 311 (3.0%) | 81 (3.8%) | 27 (3.0%) | 38 (6.4%) | 8 (12.9%) | 46 (7.2%) |
| Some difficulty | 58 (0.6%) | 28 (1.3%) | 12 (1.3%) | 26 (4.4%) | 1 (1.6%) | 21 (3.3%) |
| Extreme difficulty | 2 (0.0%) | 2 (0.1%) | 1 (0.1%) | 4 (0.7%) | 0 (0.0%) | 1 (0.2%) |
| Missing | 41 (0.4%) | 13 (0.6%) | 10 (1.1%) | 3 (0.5%) | 0 (0.0%) | 5 (0.8%) |
| Difficulty watching TV |  |  |  |  |  |  |
| No difficulty | 9268 (90.9%) | 1811 (84.5%) | 783 (86.0%) | 466 (79.0%) | 44 (71.0%) | 495 (77.1%) |
| Little difficulty | 760 (7.5%) | 249 (11.6%) | 98 (10.8%) | 89 (15.1%) | 15 (24.2%) | 104 (16.2%) |
| Some difficulty | 110 (1.1%) | 64 (3.0%) | 17 (1.9%) | 29 (4.9%) | 2 (3.2%) | 37 (5.8%) |
| Extreme difficulty | 1 (0.0%) | 2 (0.1%) | 1 (0.1%) | 1 (0.2%) | 0 (0.0%) | 1 (0.2%) |
| Missing | 61 (0.6%) | 17 (0.8%) | 11 (1.2%) | 5 (0.8%) | 1 (1.6%) | 5 (0.8%) |
| Difficulty reading newspaper/magazine |  |  |  |  |  |  |
| No difficulty | 8347 (81.8%) | 1567 (73.1%) | 683 (75.1%) | 372 (63.1%) | 31 (50.0%) | 410 (63.9%) |
| Little difficulty | 1370 (13.4%) | 408 (19.0%) | 163 (17.9%) | 130 (22.0%) | 23 (37.1%) | 140 (21.8%) |
| Some difficulty | 356 (3.5%) | 139 (6.5%) | 46 (5.1%) | 69 (11.7%) | 7 (11.3%) | 74 (11.5%) |
| Extreme difficulty | 33 (0.3%) | 13 (0.6%) | 8 (0.9%) | 14 (2.4%) | 0 (0.0%) | 15 (2.3%) |
| Missing | 94 (0.9%) | 16 (0.7%) | 10 (1.1%) | 5 (0.8%) | 1 (1.6%) | 3 (0.5%) |
| Difficulty reading food/medication labels |  |  |  |  |  |  |
| No difficulty | 4354 (42.7%) | 647 (30.2%) | 338 (37.1%) | 166 (28.1%) | 14 (22.6%) | 156 (24.3%) |
| Little difficulty | 3936 (38.6%) | 833 (38.9%) | 359 (39.5%) | 190 (32.2%) | 21 (33.9%) | 234 (36.4%) |
| Some difficulty | 1615 (15.8%) | 536 (25.0%) | 167 (18.4%) | 161 (27.3%) | 22 (35.5%) | 184 (28.7%) |
| Extreme difficulty | 261 (2.6%) | 123 (5.7%) | 40 (4.4%) | 71 (12.0%) | 5 (8.1%) | 65 (10.1%) |
| Missing | 34 (0.3%) | 4 (0.2%) | 6 (0.7%) | 2 (0.3%) | 0 (0.0%) | 3 (0.5%) |
| Difficulty going up/down steps in dim light |  |  |  |  |  |  |
| No difficulty | 8053 (79.0%) | 1492 (69.6%) | 637 (70.0%) | 350 (59.3%) | 34 (54.8%) | 370 (57.6%) |
| Little difficulty | 1683 (16.5%) | 487 (22.7%) | 183 (20.1%) | 132 (22.4%) | 21 (33.9%) | 161 (25.1%) |
| Some difficulty | 399 (3.9%) | 149 (7.0%) | 75 (8.2%) | 94 (15.9%) | 6 (9.7%) | 94 (14.6%) |
| Extreme difficulty | 32 (0.3%) | 9 (0.4%) | 9 (1.0%) | 10 (1.7%) | 1 (1.6%) | 14 (2.2%) |
| Missing | 33 (0.3%) | 6 (0.3%) | 6 (0.7%) | 4 (0.7%) | 0 (0.0%) | 3 (0.5%) |

Supplementary Table 5: Mental and physical function according to self-reported eye condition (n=14,547, excludes 45 participants with missing data on eye conditions)

|  | **Self-reported ocular condition**, n (%) | | | | | |
| --- | --- | --- | --- | --- | --- | --- |
|  | **None of these** | **Cataracts** | **Glaucoma** | **Macular degeneration** | **Retinopathy** | **>1 of these** |
|  | n=10,200 | n=2,143 | n=910 | n=590 | n=62 | n=642 |
| CES-D10 score (self-reported depression) |  |  |  |  |  |  |
| 0-7 (no/mild depressive symptoms) | 9,309 (91.3%) | 1,905 (88.9%) | 822 (90.3%) | 533 (90.3%) | 51 (82.3%) | 568 (88.5%) |
| 8-30 (depressive symptoms) | 889 ( 8.7%) | 238 (11.1%) | 88 ( 9.7%) | 56 ( 9.5%) | 11 ( 17.7%) | 73 (11.4%) |
| Missing | 2 ( 0.0%) | 0 ( 0.0%) | 0 ( 0.0%) | 1 ( 0.2%) | 0 ( 0.0%) | 1 ( 0.2%) |
| SF-12 mental component score |  |  |  |  |  |  |
| ≥60 (better function) | 3,100 (30.4%) | 606 (28.3%) | 252 (27.7%) | 156 (26.4%) | 16 (25.8%) | 199 (31.0%) |
| 57-<60 | 2,696 (26.4%) | 549 (25.6%) | 235 (25.8%) | 155 (26.3%) | 12 (19.4%) | 129 (20.1%) |
| 52-<57 | 2,078 (20.4%) | 453 (21.1%) | 197 (21.6%) | 136 (23.1%) | 13 (21.0%) | 127 (19.8%) |
| <52 (poorer function) | 2,322 (22.8%) | 535 (25.0%) | 226 (24.8%) | 142 (24.1%) | 21 (33.9%) | 187 (29.1%) |
| Missing | 4 ( 0.0%) | 0 ( 0.0%) | 0 ( 0.0%) | 1 ( 0.2%) | 0 ( 0.0%) | 0 ( 0.0%) |
| SF-12 physical component score |  |  |  |  |  |  |
| ≥55 (better function) | 2,884 (28.3%) | 526 (24.5%) | 214 (23.5%) | 132 (22.4%) | 11 (17.7%) | 141 (22.0%) |
| 50-<55 | 2,582 (25.3%) | 527 (24.6%) | 222 (24.4%) | 149 (25.3%) | 9 (14.5%) | 126 (19.6%) |
| 43-<50 | 2,339 (22.9%) | 553 (25.8%) | 247 (27.1%) | 153 (25.9%) | 20 (32.3%) | 154 (24.0%) |
| <43 (poorer function) | 2,391 (23.4%) | 537 (25.1%) | 227 (24.9%) | 155 (26.3%) | 22 (35.5%) | 221 (34.4%) |
| Missing | 4 ( 0.0%) | 0 ( 0.0%) | 0 ( 0.0%) | 1 ( 0.2%) | 0 ( 0.0%) | 0 ( 0.0%) |
| Frailty score |  |  |  |  |  |  |
| Not frail | 6,513 (63.9%) | 1,333 (62.2%) | 559 (61.4%) | 341 (57.8%) | 30 (48.4%) | 338 (52.6%) |
| Pre-frail | 3,539 (34.7%) | 779 (36.4%) | 327 (35.9%) | 234 (39.7%) | 31 (50.0%) | 287 (44.7%) |
| Frail | 148 ( 1.5%) | 31 ( 1.4%) | 24 ( 2.6%) | 15 ( 2.5%) | 1 ( 1.6%) | 17 ( 2.6%) |
| Number of falls in the past year |  |  |  |  |  |  |
| None | 7,308 (71.6%) | 1,474 (68.8%) | 600 (65.9%) | 399 (67.6%) | 38 (61.3%) | 412 (64.2%) |
| 1 | 1,495 (14.7%) | 332 (15.5%) | 167 (18.4%) | 101 (17.1%) | 6 ( 9.7%) | 109 (17.0%) |
| 2 | 800 ( 7.8%) | 187 ( 8.7%) | 81 ( 8.9%) | 47 ( 8.0%) | 13 (21.0%) | 56 ( 8.7%) |
| 3 | 324 ( 3.2%) | 79 ( 3.7%) | 33 ( 3.6%) | 26 ( 4.4%) | 2 ( 3.2%) | 33 ( 5.1%) |
| ≥4 | 179 ( 1.8%) | 53 ( 2.5%) | 20 ( 2.2%) | 11 ( 1.9%) | 3 ( 4.8%) | 20 ( 3.1%) |
| Missing | 94 ( 0.9%) | 18 ( 0.8%) | 9 ( 1.0%) | 6 ( 1.0%) | 0 ( 0.0%) | 12 ( 1.9%) |
